# Bovine Spongiform Encephalopathy  – A Review from the Perspective of Food Safety

**DOI:** 10.14252/foodsafetyfscj.2018009

**Published:** 2019-06-13

**Authors:** Susumu Kumagai, Takateru Daikai, Takashi Onodera

**Affiliations:** 1 Research Center for Food Safety, The University of Tokyo, Yayoi 1-1-1, Bunkyo-ku, Tokyo 113-8657, Japan; 2 Food Safety Commission of Japan Secretariat, Akasaka Park Bld. 22F, Akasaka 5-2-20, Minato-ku, Tokyo 107-6122, Japan; 3 Cooperative Department of Veterinary Medicine, Graduate School of Veterinary Sciences, Iwate University, Morioka-shi, Iwate 020-8550, Japan

**Keywords:** BSE, atypical, vCJD, control measure, origin

## Abstract

Bovine spongiform encephalopathy (BSE) is a fatal neurodegenerative disease that belongs to transmissible spongiform encephalopathy (TSE). Since the first case was identified in the UK in 1986, BSE spread to other countries including Japan. Its incidence peaked in 1992 in the UK and from 2001 to 2006 in many other countries, but a feed ban aimed at eliminating the recycling of the BSE agent and other control measures aimed at preventing food and feed contamination with the agent were highly effective at reducing the spread of BSE. In 2004, two types of atypical BSE, H-type BSE (H-BSE) and L-type BSE (L-BSE), which differ from classical BSE (C-BSE), were found in France and Italy. Atypical BSE, which is assumed to occur spontaneously, has also been detected among cattle in other countries including Japan. The BSE agent including atypical BSE agent is a unique food-safety hazard with different chemical and biological properties from the microbial pathogens and toxic chemicals that contaminate food. In this review, we summarize the reported findings on the tissue distribution of BSE prions in infected cattle and other aspects of BSE, as well as the control measures against the disease employed in Japan. Topics that require further studies are discussed based on the summarized findings from the perspective of food safety.

## 1. Introduction

Bovine spongiform encephalopathy (BSE) is a fatal neurodegenerative disease in bovines that belongs to transmissible spongiform encephalopathies (TSE). The first case was identified in the UK in 1986^1^^)^. The UK epidemic was attributed to the exposure to an infectious agent in 1981-1982 in association with a dramatic reduction in the use of organic solvents in the manufacture of meat and bone meal (MBM)^2^^)^. The annual number of BSE cases reported in the UK increased from 446 in 1987, to 37,280 in 1992, and then decreased to less than 10 after 2010. The number peaked in 2001-2002 in many European countries, excluding the UK, and in 2006 in Japan (World Organisation for Animal Health (OIE) up to 2016 December 31). A “feed ban” that was introduced in BSE affected countries to eliminate the recycling of the BSE agent was effective at reducing the incidence of BSE.

In 1990, surveillance of Creutzfeldt-Jakob disease (CJD) was reinstituted in the UK to identify changes in the pattern of CJD associated with the spread of BSE. CJD with a new neuropathological profile and unusual include of the young age was identified and named new variant of CJD (vCJD), raising a concern about a link of this human disease to BSE^3^^)^. Comparisons of glycoform patterns of Western blot (WB) and artificial transmissibility of BSE to experimental animals including macaques supported the link between the two diseases^4^^–^^7^^)^. A World Health Organization (WHO) Consultation held on April 2-3, 1996, concluded that although no definitive link had been identified between BSE and v-CJD, exposure to the BSE agent might be the most likely cause of vCJD. The Consultation recommended to minimize the transmission of BSE among animals and to reduce human exposure to the BSE agent as much as possible^8^^)^. The reported number of definite and probable vCJD cases from 1995 to 2014 was 177, 27, and 25, in the UK, France, and other countries, respectively^9^^)^, and 2 cases were newly reported in 2016^10^^)^.

The disease-associated isoform of the prion protein (PrP^Sc^) was detected in the tonsils, spleens and lymph nodes obtained from vCJD patients at necropsy, and in an appendix tissue removed from a vCJD patient 8 months before the onset of the disease^11^^,^^12^^)^. A large-scale survey of appendix samples that had been removed during operations in the UK showed that 16 specimens out of 32,441 appendix samples were positive for the abnormal prion protein, indicating that vCJD has a prevalence of 493 per million in the UK^13^^)^.

Prions isolated from BSE cattle were regarded to be a single strain of transmissible spongiform encephalopathy (TSE) prions^14^^)^ before atypical BSE was newly identified. Two types of atypical BSE, which had different neuropathological and molecular features from previous cases of BSE, were identified by active surveillance in 2004 in Italy and France, and named bovine amyloidotic spongiform encephalopathy (BASE, which was later also called L-type BSE (L-BSE)) and H-type BSE (H-BSE)^15^^–^^18^^)^. Since then the type of BSE that was recognized as a causative agent of the epidemic in the UK and other countries has been called classical BSE (C-BSE) to distinguish it from the atypical BSE. Compared with the un-glycosylated disease-associated isoform (PrP^d^) derived from C-BSE animals, PrP^d^ from the H-BSE and L-BSE had a higher and lower molecular mass, respectively. One hundred thirty-five atypical BSE cases (the number as of November 2018 reported by the Food Safety Commission of Japan (FSCJ)) have been identified worldwide mostly by active surveillance programs for fallen stock, and regularly and emergently slaughtered animals. The clinical symptoms of the atypical BSE observed in intraspecies transmission experiments^19^^–^^22^^)^ included an overreaction to external stimuli, unexpected startle responses, apprehension, anxiety, difficulty of getting up, and dullness, which were difficult to differentiate from the symptoms of C-BSE.

BSE PrP^Sc^ is transmissible (infectious) in various animal species and highly resistant to chemical and physical treatments^23^^)^ compared with microbial pathogens. Although transmissible prions cannot principally proliferate in/on foods composed of dead organisms, they can proliferate in living host animals. Such unique characteristics, which are distinct from those of the microbial pathogens and toxic chemicals that contaminate foods, may require specific risk-reduction measures that differ from those used for other food-safety hazards. In this review, we summarize the accumulated findings on BSE and discuss future research needs from the perspective of food safety.

Regarding the term relating to prions and BSE, we use principally the term (see “Abbreviation”) as used by the original authors when referring to published articles. The term, the BSE agent or C- or L-BSE agent, is used mainly as the material derived from BSE-infected animals.

## 2. Oral Doses and Incubation Period in Cattle

The incidence of BSE and incubation period in infected cattle were studied in the UK under the condition simulated to natural infection with the BSE agent, and the interim results were reported in 2007^24^^)^.

In one of the experiments, 10 calves at approximately 4-6 months of age were orally inoculated once with a pooled homogenate of the brainstem from BSE-affected cattle (infectious titer measured using RIII mice is 10^3.5^ intracerebral and intraperitoneal (ic/ip) 50% infectious dose (ID_50_/g)) at a dose of 100 g, 10 g or 1 g brainstem, or repeatedly with the same homogenate at a dose of 100 g per once a day for three days. In the other experiment, 15 calves were orally inoculated once with the pooled homogenate at a dose of 100 mg, 10 mg, or 1 mg, and five calves were at a dose of 1 g.

Clinical signs were detected first at 31 months post exposure in the animals challenged with 100 g of the brainstem, while the signs were detected first at 45 months post exposure in the animals challenged with 1 g of the brainstem.

From the results, one oral cattle ID_50_ was estimated to be almost equivalent to 10^2.8^ ic/ip RIII mouse ID_50_. The inoculated brainstem at a dose of 0.20 g (95% confidence interval: 0.04 to 1.00 g) was estimated to affect clinically 50% of cattle. The incidence rate (the number of BSE positive animals/the number of exposed animals) decreased with the decrease in dose, although large variations were noted in incubation period among the cattle received the same dose. The average incubation period was reduced with the increase in dose. Onset of BSE was observed even in an animal inoculated with the lowest dose (1 mg of the brainstem from BSE cattle)

The completed data of this study were reported in 2012, showing additional two BSE cases, one given 1 mg brainstem and the other given 10 mg brainstem. The incubation periods, which were determined based on definitive diagnosis of BSE, were 33-45 (n = 10), 31-60 (n = 10), 41-72 (n = 7), 45-73 (n = 10), 53-98 (n = 7), 56-109 (n = 2), and 68 months (n = 1), in animals given 3 × 100 g, 100 g, 10 g, 1 g, 100 mg, 10 mg, and 1 mg, respectively, of the BSE brainstem. Based on the result, one oral cattle ID_50_ was revised to 0.15 g of the brainstem of the BSE-affected cattle^25^^)^.

Epidemiological studies on BSE in the UK have also indicated incubation periods in natural BSE cases. The estimated average incubation period was 2.5 to 8 years^26^^)^, 4.75 to 5.00 years^27^^)^, or 5.5 years^28^^)^.

Based on the incubation period of 5 to 5.5 years estimated from data of natural BSE cases in the UK, Wells et al estimated the amount of PrP^Sc^ ingested by these cases to be 0.1 g to 1 g in terms of a single dose of the brainstem derived from the UK BSE cases, despite a large variation of the incubation period among the experimentally infected calves^24^^)^. Together with the finding of age-dependent tissue distribution of PrP^Sc^, the estimated amount of ingestion was used for the risk assessment that was required for resetting the age of cattle for SRM removal and BSE testing at slaughter in Japan (see “8. The occurrence of BSE in cattle and BSE control measures employed in Japan”). The assessment provided a basis of the revision of control measures in Japan under the situation of the lack of data of the amount of PrP^Sc^ consumed by cattle in Japan.

The dose-related timing when PrP^Sc^ could be detected in the central nervous system (CNS) and related peripheral nerve ganglions in cattle was estimated by a logistic regression analysis on the data of the cattle that were orally inoculated with the brainstem from BSE-affected cattle^29^^–^^33^^)^. The timing of detection of PrP^Sc^ was estimated to be the time point elapsed 79% and 97% of the incubation period after exposure to 100 g and 1 g, respectively, in 50% of the exposed animals. Taken together with the estimate of incubation period in the cases of the BSE epidemic in the UK, the exposure to 1 g of the brainstem from the UK BSE cases was regarded to be relevant to field situations in the UK^31^^)^. Consistent with this was the opinion of the European Food Safety Authority (EFSA) that the incubation periods of the 1 g dose group were more relevant to field situations compared to the periods of the 100 g dose group, based on the relationship between the dose and incubation period, and on the EU regulations for specific risk material (SRM) and feed^34^^)^.

Histological studies demonstrated that vacuolar degeneration appeared in the brainstem at 32- and 66-months post exposure in the 100 g dose group and 1 g dose group, respectively. PrP^Sc^ was detected first in the CNS at 30- and 44-months post exposure in the 100 g- and 1 g-dose groups, respectively. Prior to these time points, PrP^Sc^ was not detected in any nervous tissues examined^35^^)^.

Western blot (WB) analysis revealed accumulation of detectable PrP^Sc^ in the brainstem, cervical and thoracic spinal cords, and cervical dorsal root ganglia (DRG) at 32 months post exposure, in the thoracic DRG at 35 months, in the phrenic nerve and adrenal gland at 35 to 36 months, and in the sciatic nerve and stellate ganglion at 36 months in the 100 g dose cattle. In the 1 g dose group, PrP^Sc^ was detected in the midbrain, thoracic DRG, cervical and thoracic spinal cords, and sciatic nerve at 44 months, but not before 44 months. Clinical signs were observed first at 35 months and 44 months in the 100 g- and 1 g-dose groups, respectively^36^^)^.

These studies suggested that the calves orally exposed to 100 g brain tissues from BSE cases accumulated detectable levels of PrP^Sc^ in the CNS around 30 months post exposure. The calves showed clinical signs around 35 months with detectable accumulation of PrP^Sc^ in the peripheral nervous system (PNS). The timing of the PrP^Sc^ accumulation in the CNS and appearance of clinical signs were 44-months post exposure in 1 g dosed calves, indicating the delay of the timing at lower doses.

## 3. Intestinal Uptake of the C-BSE Agent

The study of *in vitro* incubation of brain homogenates from scrapie sheep with sheep alimentary fluids (rumenal, abomasal, and ileal ﬂuids, and bile) demonstrated that both normal and abnormal prions were digested readily by the alimentary fluids^37^^)^. Degradation of proteinase K (PK) resistant prion protein (PrP^res^) by intestinal contents was also observed in *in vitro* study of incubation of C-BSE brain homogenates with the gut content from sheep^38^^)^. However, changes in infectivity of the C-BSE agent remained uncertain, because only IHC and WB were used to detect PrP^res^ in those experiments. Scherbel et al found also the decrease in scrapie PrP^Sc^ to the level undetectable by WB after incubation of scrapie-infected hamster brain homogenates with bovine ruminal and colonic homogenates, but they found no changes in infectivity of the incubated brain homogenates^39^^)^. A study of incubation of C-BSE brain homogenates with bovine ruminal and colonic homogenates suggested that C-BSE PrP^Sc^ was not degraded in the bovine gastrointestinal tract^40^^)^. These findings on degradation of PrP^Sc^ in the intestinal lumen suggest that C-BSE PrP^Sc^ is stable in the lumen. However, there are no data that demonstrated directly the intestinal degradation of atypical BSE PrP^Sc^, which was more sensitive against PK digestion than C-BSE PrP^Sc41,42)^.

PrP^Sc^ is accumulated in the gut-associated lymphoid tissues (GALT) of Payer’s patches (PPs). PrP^Sc^ may be taken up by M cells on PP or/and by villus epithelial cells from intestinal lumen. The involvement of M-cells has been demonstrated by *in vivo* experiments using mice of which M cells were depleted or enhanced^43^^,^^44^^)^, and an *in vitro* experiment using bovine M cell model^45^^)^.

Regarding prion uptake by villus epithelial cells, uptake by endocytosis of BSE PrP^Sc^ via 37 kDa/67 kDa laminin receptor has been observed in human enterocytes^46^^)^. Using high-resolution immunofluorescence and cryo-immunogold electron microscopy, Kujala et al detected PrP in large endosomes of enterocytes in the follicle-associated epithelium and at much lower levels in M cells in the mice, which were inoculated with the brain homogenate from scrapie-affected mice. Immunolabeled PrP was also detected on plasma membranes of follicular dendritic cells (FDCs) in germinal centers of PP^47^^)^. Ackerman et al found accumulation of BSE prion (PrP^BSE^) in tingible body macrophages (TBMs) and FDCs in ileal PP of the calves orally inoculated with the brain from C-BSE cattle^48^^)^. Out of 53 calves that showed immunolabeling in GALT after receiving oral inoculation of the brainstem from C-BSE cases, 22 (41.5%) had TBM labeling only, while 31 (58.5%) had TBM and FDC labeling. Labeling of the enteric nervous system was observed in 2 cases^49^^)^.

Thus, after being taken up by M cells on PP or/and by villus epithelial cells from the intestinal lumen, PrP^Sc^ is transported to enteric nerves via TBM and FDC, and then to the CNS through sympathetic and parasympathetic routes. From the CNS, PrP^Sc^ may spread centrifugally to peripheral nerves^50^^–^^52^^)^.

Based on reported weight of PP in beef cattle and the risk of BSE infection that was estimated from the data of BSE cases in British cattle, St Rose et al found an association between PP development and susceptibility of cattle to BSE infection (Spearman’s rank correlation coefficient = 0.693 (n = 94, *P* < 0.001)). The weight of PP tissue in the small intestine increases in the first year of life, peaks at 12–18 months of age, declines thereafter, while the risk of infection is initially low, peaks at about 12 months, and then declines rapidly^53^^)^.

In the calves orally inoculated with a pooled homogenate of the brainstem from C-BSE cattle, total numbers of follicles in the intestine decreased with the increase in cattle age. The probability of finding of the follicles decreased by approximately 5.2%, 6.2% and 4% in the duodenum, jejunum and ileum, respectively, for each 1-month increase of cattle age^49^^)^. In agreement with this, involution of the follicles was observed from 10 months old, and hypocellular and shrunken follicles were from 32 months old, in calves received oral inoculation of C-BSE brainstem. In the calves, immunostaining existed in follicles of ileal continuous PP from 6- to 40-months post exposure, but not at 2- and 32-months post exposure^54^^)^.

Sheep were more susceptible to orally inoculated C-BSE brain at their ages less than 12 hr or at 2-3 months than at their ages of 3, 6, and 15-27 months^55^^)^. In sheep, ileal PP (IPP) begins to involute from about 12 weeks old and only a few PP follicles remain in the ileum at 18 months old^56^^)^.

Taken together with the findings in sheep, susceptibility of calves to oral C-BSE PrP^Sc^ is likely to decrease with the increase in their age in association with the reduction of the IPP-mediated transfer of the PrP^Sc^ from the intestinal lumen to the enteric nerves. To confirm this notion and the quantitative age-susceptibility relationship, however, direct evidence is required for the age-dependent susceptibility of cattle to the C-BSE agent.

## 4. Tissue Distribution of PrP^Sc^ in Cattle Experimentally Exposed to C-BSE

Understanding of distribution of PrP^Sc^ in cattle tissues is the base for the prevention of ingestion of the BSE agent via food as is for marine toxins in finfish and shellfish tissues, and various toxic alkaloids in plant tissues. The distribution of PrP^Sc^ has extensively been studied in cattle orally inoculated with the BSE agent. Infectivity of tissues, dose-related incidence of infection, and incubation period have also been studied by experimental oral inoculation of the BSE agent to cattle.

Infectivity of cattle tissues has been studied by bioassays using calves, wild-type mice such as RIII mice, or transgenic (Tg) mice expressing bovine or other species’ prions. According to Wells et al^24^^)^, one cattle oral ID_50_, is equivalent to 10^2.8^ RIII mouse ic/ip ID_50_, while one RIII mouse ic/ip ID_50_, is equivalent to 10^2.7^ cattle ic ID_50_. One oral cattle ID_50_, therefore, is equivalent to 10^5.5^ cattle intracerebral ID_50_. The experiment of titration conducted by Buschmann and Groschup demonstrated that titers of the same brainstem pool from cattle were 10^3.3^ and 10^7.7^ ic/ip ID_50_/g in RIII mice and in Tg mice overexpressing bovine normal cellular isoform of prion protein (PrP^C^) (Tgbov XV mice), respectively, indicating the orally inoculated cattle to be approximately 7 logs less susceptible than the Tgbov XV mice received ic/ip inoculation. Therefore, bioassays using ic/ip exposure to Tg mice (Tgbov XV mice), ic exposure to cattle, and ic/ip exposure to wild mice (RIII mice) are more sensitive in this order^57^^,^^58^^)^.

Large-scale experiments of infection of cattle with the BSE agent were performed by the UK researchers. The brainstem (infectious titer per g was approximately 10^3.1-3.5^ RIII mice ic/ip ID_50_) from the BSE cases identified in the UK was orally inoculated to calves aged 4-6 months.

In the group exposed to 100 g of the BSE brainstem, PrP^Sc^ was not detected in the brain at 22, 26, and 27 months post exposure, but detected first at 30 and 32 months with or without clinical signs^31^^,^^49^^,^^59^^)^.

PrP^Sc^ was detected by IHC in the jejunum and ileum from 4 months post exposure, which is earlier than the time of detection in the CNS, until 30 and 42 months, respectively, but not detected in the duodenum during the same period. The rate of detection of PrP^Sc^ (the number of positive cattle over the number of tested cattle) during the entire period was lower in the jejunum (8/58, 13.8%) than in the ileum (45/99, 45.5%). In positive intestines, the average frequency of positive lymph follicle was similar in the jejunum and ileum, i.e. 1.47% and 1.26%, respectively. PrP^Sc^-positive follicles were detected in the ileum of all the cattle despite the lack of detectable PrP^Sc^ in the medulla. At 33 months post exposure, however, ileal PrP^Sc^ was not detected despite the presence of detectable PrP^Sc^ in the medulla in 2 out of 6 cattle examined. The absence of PrP^Sc^ in the ileum and its presence in the medulla were noted in 5 out of 9 cattle examined at 41-42 months and in 8 out of 10 cattle examined at 44-60 months. The number of lymph follicles in the ileum and the number of cattle having PrP^Sc^ positive lymph follicles in their intestine decreased with the increase in cattle age.

In the 1 g group, PrP^Sc^ was not detected in the brain of any animals at 42 months post exposure, while it was detected first at 44 months in the brain of one animal showing clinical signs. PrP^Sc^ was detected in ileal lymph tissues from only one animal at 24 months. PrP^Sc^ was hardly detected in the enteric nerve tissues, and not detected in the jejunum and duodenum.

The infectivity of tissues from the cattle orally exposed to 100g BSE brainstem was studied by bioassay using RIII mice (detection limit,10-1.3 RIII mouse ic/ip ID_50_ per g), which received ic/ip inoculation with pooled homogenates of various tissues from the cattle killed at each time point. The infectivity was lower in dorsal root ganglia (DRG) than in the CNS by approximately 10 RIII mouse ic/ip ID_50_ per g. The infectivity of the distal ileum was detected at 6-months post exposure, increased at 14 to 18 months, then decreased to the non-detectable level at 36 months, and increased again from 38 to 40 months. The maximum infectious titer was estimated to be 10^1.59^ RIII mouse ic/ip ID_50_ per g on average at 14 months post exposure, the value being equivalent to 10^-1.21^ cattle oral ID_50_ per g.

A notable finding of this study was similar levels of infectivity in the ileum and brain. The distal ileum at 14 months post exposure caused disease with incubation period of 455 ± 12.8 days in 15 out of 18 mice, while the caudal medulla at 40 months caused disease with incubation period of 485.0 ± 12.8 days in 11 out of 12 mice^60^^)^.

Various tissues from the BSE-exposed calves, which were killed at sequential time points from 6 to 36 months post exposure, were furthermore studied for infectivity by bioassay using calves that were intracerebrally inoculated with the tissues. The medulla oblongata and spinal cord at 6-, 10-, 18-, and 26-months post exposure showed no infectivity in the bioassay, but those taken at 32 months induced clinical signs in the bioassay calves. The infectious titer of the CNS at 32 months was estimated to be 10^-2.7^ cattle oral ID_50_. The distal ileum at 6, 10, and 18 months and the palatine tonsil at 10 months possessed infectivity in the bioassay. However, the other tissues including muscle, thymus, liver, and mesenteric lymph nodes showed no infectivity^29^^,^^58^^,^^59^^)^.

All the results demonstrate that the brain, spinal cord, DRG, and ileum are the major locations of PrP^Sc^ accumulation in experimentally-infected BSE cattle. Infectivity was higher in the ileum than in the other portions of small intestine regardless of inoculation doses^49^^)^. No data is available on the ileal infectivity at timepoints later than 40 months post-oral exposure.

To study infectivity of tissues of BSE-infected cattle, German researchers used Tg mice overexpressing bovine PrP (TgbovXV), which were 10,000 times and 10 times more susceptible to the BSE agent than RIII mice and cattle, respectively^57^^,^^61^^)^. Calves at the age of 4-6 months were orally exposed to the brainstem from BSE cases (10^6.1^ ic/ip TgbovXV mice ID_50_/g) at a dose of 100 g tissues, and sequentially killed from 1- to 44-months post exposure.

The calves showed clinical signs first at 32 months post exposure. PrP^Sc^ in the brain was not detected by IHC until 20 months, but that was detected at 24 months^50^^,^^61^^)^. PrP^Sc^ was detected in the ileum and ileocaecal-junction from 4 to 44 months and from 12 to 24 months, respectively, by IHC, enzyme-linked immunosorbent assay (ELISA), and WB, but PrP^Sc^ was not detected in any parts of the small intestine at 1 month and in the jejunum at any time points. IHC revealed the presence of PrP^Sc^ in TBM in the ileum.

Infectivity was detected in the ileum and ileocaecal-junction at any timepoints from 8 to 20 months post exposure with the highest level at 12 months. Infectivity was not detected in these intestinal tissues at 24 months, but the infectivity at later stages remains unclear. The jejunum had infectivity, although its potency was much lower than that in the ileum.

In the other study, the calves that were orally exposed to 100 g BSE brainstem were also examined for PrP^Sc^ deposits and infectivity in their tissues from 16 to 44 months post exposure by IHC and bioassay using TgbovXV Tg mice. PrP^Sc^ was detected at the obex in one of the two calves at 28 months, but not in any calves at 24 months. However, the caudal medulla at 24 months showed weak infectivity in one of the 7 inoculated mice. PrP^Sc^ was detected in lymph follicles and intestinal nerve system in the distal ileum at any timepoints (infectivity of the distal ileum was not shown in this study). Infectivity was detected in sympathetic and parasympathetic nerve ganglia at 16 and 20 months, although PrP^Sc^ was not detected in these tissues. Also, the transient presence of infectivity was noted in the thoracic spinal cord (T7) at 16 months, although PrP^Sc^ was not detected^62^^)^. In an asymptomatic calf killed at 24 months post exposure, PrP^Sc^ was detected by IHC in the spinal cord, ileum, medulla oblongata, the bridge, celiac ganglion, and caudal mesenteric ganglion, but not detected in gut-associated lymphoid tissues including the tonsil, jejunal lymph nodes, ileal lymph nodes, splenic lymph nodes, colic lymph nodes, and spleen. The results suggested that in some individuals exposed to a high oral dose, PrP^Sc^ reached their brain within 24 months via a route from the intestinal membrane ganglion complex through the nerves of viscera and lumbar/caudal thoracic spinal cord (sympathetic innervation of digestive canal) or via a route running through vagal nerve (parasympathetic innervation of digestive canal)^50^^)^. The earliest time point of detection of PrP^BSE^ and infectivity of the IPP was investigated in the calves killed at 1 week, and 2-, 4-, 6- and 8-months post oral exposure. The infectivity was examined by bioassay using TgbovXV Tg mice, and PrP^BSE^ was by IHC and protein misfolding cyclic amplification (PMCA) assays. Infectivity and PrP^BSE^ were detected first in PP at 1- week post exposure. IHC revealed the presence of PrP^BSE^ in FDCs of PP follicles at 4 months post exposure. These data indicated that PrP^Sc^ was propagated in IPP within 2 months of oral exposure to the BSE agent^48^^)^.

Tissues from 4 cattle, which were orally exposed to 100 g brainstem from BSE cattle and killed in various clinical stages of disease from 36- to 50-months post exposure, were examined for the presence of PrP^Sc^ by PMCA (an analytical sensitivity was comparable to Tg mouse bioassay, allowing detection of the 10^6^ dilution of a 10% BSE-positive brain homogenate with infectivity titer of 10^6.1^ mouse LD_50_/g). PrP^Sc^ was detected not only in the brain, spinal cord, nerve ganglia, optic nerve, mesenteric lymph nodes, PP, and adrenal glands, but also in the esophagus, rumen, abomasum, and rectum. However, PrP^Sc^ was not detected in the biceps brachii muscle, longissimus dorsi muscle, psoas major muscle, semitendinosus muscle, heart, lung, liver, gall bladder, pancreas, kidney, spleen, and tonsil^63^^)^.

In studies performed by Japanese researchers, calves aged 3 to 11 months were orally inoculated with a pooled homogenate of the brainstem from BSE cases at a dose of 5 g brainstem. Infectious titer of the inocula was approximately 10^6.7^ ic LD50/g in bovine PrP-overexpressing Tg mice (TgBoPrP mice)^64^^,^^65^^)^, which were 10-fold or 1000-fold more susceptible compared to cattle or RIII mice, respectively^66^^)^. PrP^Sc^ was detected by WB and IHC in the CNS from 34 months post exposure, but not detected at 30 months^67^^)^.

In their studies, the small intestine of 3 meters length from the ileocecal junction, the location containing both single-large continuous PP (CPP) and discrete PPs (DPPs), was examined for the presence of PrP^Sc^ at 50 cm length intervals^67^^)^. Cattle have a CPP of approximately 2–3 m length along the posterior jejunum and entire ileum, DPPs scattering in the jejunum as 30–40 lymphoid aggregates, and both CPP and DPP in the posterior portion of the jejunum^68^^,^^69^^)^. PrP^Sc^ was detected in CPP in 5 asymptomatic calves (3 at 20 months, 1 at 30 months and 1 at 46 months post exposure) among the 28 inoculated calves, but not in any of the 9 calves at 48-72 months. PrP^Sc^ was not detected in DPP in all the calves at any time points. PrP^Sc^ positive cells in the follicles were identified as TBM. Infectivity was detected in the CPP at 20 months post exposure with 248.9 ± 14.4 days of incubation period in the Tg mice. The DPP-inoculated mice showed no signs of infection for 650 days. These results indicate that intestinal risk is concentrated in CPP^67^^)^.

Being consistent with this notion, a study of intestinal uptake of various particles such as carbon black, fluorescein isothiocyanate (FITC)-labeled latex, FITC-labeled dextran, bovine serum, and recombinant mouse prion protein suggested more effective absorption of particles in distal IPP than DPPs in newborn and 2-months old calves. Moreover, intestinal absorption of the particles was more efficient in newborn than in 2-months old calves, indicating that BSE PrP^Sc^ could be more effectively absorbed from the intestine at neonatal ages than other ages^69^^)^.

PrP^Sc^ was detected by WB in several peripheral nervous tissues and the adrenal gland in experimentally affected cows at the terminal disease stage. PrP^Sc^ was furthermore detected by serial potassium dextran sulfate-PMCA in the palatine tonsils, lymph nodes, ileocecal region, and muscle, but not detected in the spleen, blood, and cerebrospinal fluid (CSF). The level of PrP^Sc^ estimated from the amplification factor in PMCA was 10^6^ -fold lower in muscle tissue than in the brain^70^^)^.

Okada et al detected PrP^Sc^ using IHC in the muscle of BSE-infected clinically affected cattle, but not in the preclinical cattle. A small amount of PrP^Sc^ deposits was also detected in the muscle spindle^71^^)^, but its quantitative contribution to PrP^Sc^ in a whole muscle is unknown.

Espinosa et al studied infectivity of tissues of BSE-infected asymptomatic cattle, which were orally inoculated at the age of 4 to 6 months with 100 g of the brainstem from BSE-affected cattle and killed at 20-, 24-, 27-, 30-, or 33-months post inoculation. The infectivity was detected first in the brainstem at 27 months by bioassay using Tg mice overexpressing bovine PrP (BoPrP-Tg110 mice). The infectivity was also noted in the sciatic nerve at 30 and 33 months, and in PPs and tonsils at any time points, although lower than that in the brain. The spleen, skeletal muscle, blood and urine showed no detectable infectivity. From these findings, the authors concluded that PrP^Sc^ propagates through the nervous system in asymptomatic cattle^72^^)^.

The UK study showed that fat tissues from the cattle orally inoculated with 100g brainstem of BSE-affected cattle did not cause infection to wild-type mice^73^^)^. Although infectivity in mice was detected in the perirenal, perimuscular, omental, and mediastinal fat of chronic wasting disease (CWD)-infected deer, and in brown fat and white fat from scrapie-infected mice, the infection titer was far below that of the brain^74^^,^^75^^)^.

## 5. Tissue Distribution of PrP^Sc^ in Natural C-BSE Cases

Infectivity was studied by bioassays using RIII mice and Tgbov XV mice for various tissues and fluids from a naturally infected C-BSE case at a late-stage of disease in Germany. Examined tissues and fluids were the brainstem, thoracic and lumber spinal cords, facial nerve, sciatic nerve, retina, optic nerve, radial nerve, cerebrospinal fluid, spleen, tonsils, distal ileum, mesenteric lymph nodes, semitendinosus muscle, longissimus dorsi muscle, heart, caruncle, amniotic fluid, and colostrum. Among these tissues and fluids, the infectivity was detected in the brainstem, and thoracic and lumber spinal cords by RIII mice bioassay, and in the retina, optic nerve, distal ileum, facial nerve, sciatic nerve, and semitendinosus muscle by the Tgbov XV mice bioassay. The authors of the study assumed that the infectivity of the semitendinosus muscle was attributable to the distribution of sciatic nerves. Estimated infectivity of the muscle was 1/10^6^ of that of the brainstem^57^^)^.

Infectivity was also studied by the TgbovXV mice bioassay for various tissues derived from two naturally infected BSE cases identified at terminal stages in the UK. The infectivity was higher in the brainstem, trigeminal ganglion, and cervical cranial ganglion than in the optic and facial nerves. The infectivity of the nasal mucosa and tongue was further lower^76^^)^.

Using IHC and WB methods, Iwata et al studied the distribution of PrP^Sc^ in various tissues in three C-BSE positive pre-clinical cattle slaughtered at their age of 80 to 95 months in Japan. Examined tissues were the liver, spleen, kidney, heart, lungs, tongue, stomach, duodenum, distal ileum, small intestine at the position of 2 or 6 meters from the distal end, cecum, rectum, colon, retina, pancreas, adrenal cortex, lymph nodes, palatine tonsil, muscle, frontal lobe, caudate nucleus, optic thalamus, corpus striatum, hippocampus, occipital lobe, cerebellar cortex, medulla oblongata, DRG, and peripheral nerves, and cervical-, thoracic-, and lumber-spinal cords. PrP^Sc^ was detected in the cerebellar cortex, medulla oblongata, and dorsal root ganglion, and cervical-, thoracic-, and lumber-spinal cords in all the animals. PrP^Sc^ was also detected in the femoral and lumber nerves (30 cm from the DRG), but at trace levels that were estimated to be 1/1,000 to 1/4,000 of the level in the spinal cord. However, PrP^Sc^ was not detected in the other tissues including the distal ileum PP and palatine tonsils^77^^)^.

In four naturally affected BSE cases found at their age of 54, 64, 69, and 102 months, PrP^Sc^ was detected by modified IHC in the jejunum and ileum containing CPP, at the location of one meter or 30 centimeters apart from the ileocecal junction. PrP^Sc^ was also detected in the colon at the 54 months old case. WB and bioassay using TgBoPrP mice, however, indicated relatively small amounts of PrP^Sc^ deposits in these intestinal tissues. No PrP^Sc^ was detected in the duodenum, jejunum with or without DPP, ileocecal junction, cecum, and rectum of any cases^78^^)^.

Taken together with the difference in sensitivity among bioassays using Tg mice, wild-type mice, and calves, all-over the findings of tissue distribution in naturally and experimentally infected BSE cattle suggest that the infectivity of tissues of the cattle is the highest in the CNS, DRG, tonsil and ileum, while relatively low in the PNS, muscle, lymph nodes, adrenal glands and jejunum, depending on the period elapsed after the exposure to the BSE agent. Infectivity of the ileum is concentrated mainly in CPP. Infectivity is further lower in other tissues.

## 6. Transmission Characteristics of Atypical BSE

The retrospective WB analysis of BSE cases in France demonstrated that the number of H- or L-BSE cases had been one or two in each annual birth cohort from 1986 through 1997, whereas the number of C-BSE cases in each birth year had increased rapidly from 1990 and reached 241 in 1995, and then decreased^79^^)^. In addition to this finding, atypical BSE cases have been reported in countries such as Norway, Romania, and Brazil, where no C-BSE cases were reported^80^^)^. Therefore, the occurrence of H-BSE and L-BSE was unlikely due to feed-mediated infection, but is likely due to sporadic nature^17^^,^^81^^)^

L-BSE can be readily transmitted to various animals by experimental infection. The experiments using bovine-PrP overexpressing Tg (Tgbov XV) mice, human-PrP overexpressing Tg mice, macaques, and cattle have suggested that the virulence of L-BSE (BASE) is higher than that of C-BSE^18^^,^^82^^–^^84^^)^. The EFSA reported that the incubation period and survival time of the L-BSE agent-inoculated mice were shorter than those of the C-BSE agent-inoculated mice, and therefore, that zoonotic potential of L-BSE PrP^Sc^ is equal to or higher than that of C-BSE PrP^Sc 82)^ .

The presence of species barrier to C-BSE between humans and bovines was suggested by the studies of experimental transmission using human- or bovine-PrP expressing Tg mice^85^^–^^87^^)^. Atypical BSE has also been studied for its transmissibility by using Tg mice. Gene-targeted Tg mice that express human PrP (HuTg mice) or bovine PrP (Bov6 mice) were intracerebrally inoculated with atypical BSE (BASE and H- BSE) isolates derived from field cases. The gene-targeted Tg mice expressing human PrP (HuMM, HuMV and HuVV corresponding to the PrP codon-129 methionine/methionine, methionine/valine, and valine/valine, respectively, in human population) at wild-type levels did not show any signs of disease, whereas the mice expressing bovine PrP showed clinical signs, indicating a transmission barrier to atypical BSE between bovines and humans^88^^)^

Sub-passage of some BASE-challenged HuTg mice caused disease in recipient Bov6 mice, but not in HuTg mice. The disease was transmitted from the apparently healthy HuTg mice to the Bov6 mice, indicating the presence of subclinical BASE infection in the HuTg mice at the level undetectable by current diagnostic methods. The lack of transmission to HuTg mice on sub-passage demonstrated lower efficiency of BASE transmission to mice expressing human PrP than to those expressing bovine PrP, and therefore suggested that humans could be less susceptible than bovines^89^^)^.

Comparative pathogenicity of C-BSE and atypical BSE has been studied by using Tg mice. Torres et al studied transmission of H-BSE, in comparison with that of C-BSE, to HuPrP-Tg340 mice overexpressing human PrP and BoPrP-Tg110 mice overexpressing bovine PrP. The brainstem homogenates from an affected C-BSE or H-BSE cow were intracerebrally inoculated to the mice for the first passage. PrP^res^-negative brain homogenates from the non-affected mice at the first passage were used for the second passage. The C-BSE isolate induced the disease with detectable PrP^res^ in both BoPrP-Tg110 and HuPrP-Tg340 mice, whereas the H-BSE isolate induced the disease only in BoPrP-Tg110 mice even at the second passage, indicating lower infectivity of H-BSE than that of C-BSE in humans^90^^)^.

Kong et al inoculated intracerebrally the brain homogenates from BASE cattle to the Tg mice that express human PrP at the wild-type level. The attack rate (60%) was higher than the reported attack rate (approximately 20%) in C-BSE-inoculated Tg mice overexpressing human PrP, indicating higher zoonotic potential of BASE than that of C-BSE^84^^)^. Beringue et al studied infectivity of H-BSE and L-BSE in comparison with C-BSE by serial ic transmission in Tg mice overexpressing human PrP (codon 129 MM). Brain homogenates from bovine cases were inoculated to the mice for the first passage, and the brain from the challenged mice was used for the second and third passages. In the mice for the first passage, L-BSE isolates induced neurologic disease in almost all the mice, whereas C-BSE isolates did not induce clear neurologic signs and H-BSE isolates did not induce the disease, indicating that L-BSE, C-BSE and H-BSE were more highly infectious in this order. The electrophoretic proﬁle of L-BSE PrP^res^ was maintained in the mice received the secondary passage^91^^)^.

However, the gene-targeted Tg mice that express the same level of bovine PrP as wild-type murine PrP showed a different feature. They were intracerebrally inoculated with brain homogenates from BASE or C-BSE cattle. Survival time was not shorter in the BASE-inoculated mice than the C-BSE-inoculated mice. Intensity of PrP deposition in the brain and severity of vacuolar degeneration were less pronounced in the BASE inoculated mice compared with the C-BSE inoculated mice. This finding did not support higher pathogenicity of the BASE agent compared with the C-BSE agent^92^^)^. Consistent with this finding, the study using Tg mice overexpressing ovine PrP showed that C-BSE was transmitted to the mice, whereas L-BSE was not, indicating lower zoonotic potential of L-BSE compared with C-BSE^93^^)^.

A study of oral inoculation of the L-BSE agent to calves^94^^)^ demonstrated that infectivity of the L-BSE agent was lower than that of the C-BSE agent. In this study, calves at the age of 3–5 months were orally inoculated with 1 g, 5 g, 10 g, or 50 g of a pooled whole-brain homogenate prepared from the cattle that were experimentally infected with L-BSE (titer; 10^6.9^ LD_50_/g in TgBo PrP mice). One of two calves given 50 g of the homogenate showed clinical symptoms at 88 months post exposure, but the other was clinically healthy until 94 months post exposure. The other doses induced neither clinical signs nor detectable PrP^Sc^ at 51- to 86-months post exposure, indicating lower oral infectivity of L-BSE than C-BSE in cattle (a dose of 1 mg brainstem from C-BSE affected cattle induced the disease^25^^)^, see “2. Oral doses and incubation period in cattle”) . The difference in oral infectivity to cattle between C-BSE and L-BSE is consistent with the epidemic of C-BSE and sporadic occurrence of L-BSE in cattle fed MBM.

The observed orally effective dose (50 g) and that often used in ic transmission experiments (1 ml of 10% brain homogenate)^20^^–^^22^^,^^95^^)^ may indicate that cattle are much less susceptible to oral than ic exposure to L-BSE. Such a difference in the effect between oral and ic inoculation has also been observed in mice inoculated with the C-BSE agent^60^^,^^96^^,^^97^^)^.

Many intra-species transmission studies demonstrated that pathological and molecular phenotypes of H-BSE- and L-BSE were maintained in cattle^20^^,^^22^^,^^95^^)^. However, these atypical strains are unstable in rodents. Changes of phenotype of L-BSE to that of C-BSE have been observed in non-Tg mice^98^^)^, Tg mice overexpressing ovine PrP^99^^)^, and Syrian hamsters^100^^)^. Balkema-Buschmann et al reported that passage of L-BSE in Tg mice overexpressing ovine PrP (TgshpXI mice) resulted in generation of PrP with a glycosylation pattern intermediate of L-BSE and C-BSE prions^101^^)^.

Changes of phenotype of H-BSE to that of C-BSE have also been observed in transmission experiments using wild-type mice. Baron et al found that H-BSE PrP^d^ changed to the PrP^d^ with similar properties as C-BSE PrP^d^ after two-generation passages in C57BL/6 wild-type mice. WB patterns of PrP^res^ in the brain were similar in the mice received the second passage of H-BSE and those of C-BSE. The third passage with this “C-BSE like” brain caused infection in recipient mice, in which “C-BSE like” PrP^res^ was maintained in the spleen^102^^)^. Torres et al found similar histopathological changes and WB patterns of PrP^Sc^ in H-BSE- and C-BSE-infected mice^103^^)^. Changes of H-BSE PrP^Sc^ to C-BSE-like PrP^Sc^ were also observed in experiments of sequential passages of H-BSE using inbred VM mouse line carrying Prn-pb (Sincp7p7)^104^^)^.

Generation of a new type of BSE was found in TgBoPrP mice that received serial passages of H-BSE. The mice showed neurological disease after the incubation period of 320.1 ± 12.2 days at the primary passage by ic inoculation of the brain from H-BSE cattle. Incubation periods in the mice received the second and third passages were shorter than the mice received the primary passage. For the fourth passage, the brain of 8 diseased mice of the third passage was each inoculated to other TgBoPrP mice. A group of the fourth-passaged mice received the brain from one of the 8 mice that showed shorter incubation periods (108.8 ±4.0 days) than the other mice. WB analysis demonstrated that PrP^Sc^ of the mice with short incubation-type of BSE (designated as BSE-SW) resembled PrP^Sc^ in C-BSE mice, but the degree of brain vacuolation and neuroanatomical distribution patterns of the BSE-SW mice were different from those of H-BSE and C-BSE mice, indicating distinct neuropathological properties of BSE-SW. Furthermore, those authors inoculated intracerebrally the brain tissue from the BSE-SW mice to three calves, which subsequently showed characteristic neurological signs of dullness after the incubation period shorter than that reported in cattle inoculated with H-BSE, L-BSE or C-BSE brain tissue. The brain of all the BSE-SW inoculated calves had severe spongiform changes, and a widespread and uniform distribution of PrP^Sc^. The transmitted BSE-SW had the biological properties distinct from those of H-BSE, but clinical and pathologic features in the transmitted cattle were indistinguishable from those of H-BSE cattle^105^^,^^106^^)^.

The transmissibility of the C-BSE agent to nonhuman primates has been observed in orally or intracerebrally inoculated lemurs (a small-sized and short-lived species *Microcebus murinus*) and cynomolgus macaques (*Macacca fascicularis*)^107^^–^^115^^)^. According to Lasmézas, the results so far show that 5 g of the infectious C-BSE cattle brain is enough to induce the disease in all the recipient animals via oral route, with 500 mg yielding an incomplete attack rate^116^^)^.

The transmissibility of the L-BSE agent to nonhuman primates has also been studied. Two cynomolgus macaques intracerebrally inoculated with a brain homogenate from a head of L-BSE cattle showed neurological signs and symptoms 19-20 months after inoculation. IHC revealed severe spongiform changes and PrP^Sc^ accumulation in their brain. Although the inoculated amount of PrP^Sc^ was 1/5 of that of the C-BSE brain (20 mg) used for the other experiment by the same authors^113^^)^, incubation period was 2/3 shorter in the animals inoculated with the L-BSE tissue than those with the C-BSE tissue. This may indicate that zoonotic potential of L-BSE is higher than that of C-BSE in cynomolgus macaques^117^^)^.

The brain (25 mg, mix of brainstem and thalamus) from a head of asymptomatic BASE cattle was intracerebrally inoculated to a cynomolgus macaque, and the C-BSE brainstem (100 mg, brainstem from infected UK cattle) was intracerebrally inoculated to two macaques. The BASE inoculated macaque showed shorter incubation period than the two C-BSE inoculated macaques^83^^)^. This result also indicated zoonotic potential of BASE to be higher than that of C-BSE in cynomolgus macaques, but caution may be needed for this notion because only a few animals were used in the study.

Five or 3 gray mouse lemurs were fed with the brain homogenates from L-BSE infected cattle at a dose of 5 or 50 mg, respectively. Three of the 5 fed with 5 mg and 2 of the 3 fed with 50 mg showed neurologic symptoms. In these symptomatic lemurs, PrP^res^ with a low molecular mass was detected by WB analysis as noted in L-BSE cattle, but the PrP^res^ in the lemurs contained a higher proportion of di- and monoglycosylated species than that of the bovine L-BSE^118^^)^. The effective dose in the lemurs was very low compared with that in cynomolgus macaques fed with C-BSE, which did not affect any of 6 macaques at a dose of 500 mg till 70 months^119^^)^. Oral transmission of L-BSE has not been studied in other non-human primates and available findings were mostly based on a limited number of animals, and therefore, further studies will be needed on oral infectivity of the L-BSE agent in non-human primates.

## 7. Tissue Distribution of PrP^Sc^ in Atypical BSE Cases

Distribution of PrP^Sc^ in the brain was studied in the cattle that were intracranially inoculated with the brain homogenates from natural L- and H-BSE cases. For comparison, the brain of orally challenged C-BSE cattle was analyzed. High concentrations of PrP^Sc^ were detected in the brainstem, basal nuclei, thalamus and rhinencephalon in the L-BSE inoculated cattle, in the brainstem and thalamus in the H-BSE inoculated cattle, and in the brainstem in the C-BSE inoculated cattle^42^^)^. Differences in distribution of PrP^Sc^ in the brain between atypical BSE and C-BSE cattle have also been observed in other studies^120^^)^.

The tissues from a head of clinical cattle that were intracerebrally inoculated with the brain from H-BSE cattle or from L-BSE cattle were examined for the presence of PrP^Sc^ using enzyme immunoassay in comparison with tissues from a clinical C-BSE case. In any of animals, high levels of PrP^Sc^ were found in the brain, and low levels were in the peripheral tissues including facial nerve, sciatic nerve, phrenic nerve, IPP, jejunal PP, semitendinosus muscle, tonsil, and spleen^22^^,^^121^^)^.

The PrP^Sc^ level higher in the brain than in peripheral tissues is consistent with PrP^C^ levels analyzed by quantitative WB. Levels of PrP^C^ expression are the highest in the cerebellum, obex and spinal cord, intermediate in nerve, thymus, intestine, heart and spleen, and the lowest in the lung, muscle, kidney, lymph node, skin, pancreas and liver^122^^)^.

Konold et al^19^^)^ detected PrP^Sc^ in muscles and intestines including PP from H-BSE and L-BSE cattle. In their study, the calves were intracerebrally inoculated with L-BSE or H-BSE brain homogenate at 10-11 months of age and then culled at 17-21 months post exposure in clinical end-point. Various peripheral tissues of the calves were examined for the presence of immunolabeling by IHC. Immunolabeling was found in the trigeminal ganglion, the muscle spindles of the extraocular muscles, and other muscles that contain muscle spindles, but not in the lymphoid tissues or the enteric nervous system. Brain homogenates from 2 L-BSE cases and 2 H-BSE cases used in their study were intracerebrally inoculated to the other cattle of 7 months old. After being culled at 18–22 months post exposure in clinical end-point, the recipient cattle were examined for the presence of immunolabeling in their spinal cord, mesenteric lymph node, distal ileum, palatine tonsil, medial retropharyngeal lymph node, trigeminal ganglion, extraocular muscles, and the triceps, medial gluteal and semitendinosus muscles. Among these tissues, immunolabeling was not detected in the lymphoreticular system and the peripheral tissues other than muscle spindles^19^^,^^95^^)^.

Suardi et al studied distribution of PrP^res^ in tissues of a cow, which was intracerebrally inoculated with the brain homogenate from a natural BASE case. The cow was culled at the terminal disease stage. Tissues from two asymptomatic BASE cases identified by active surveillance were also studied for PrP^res^ distribution. IHC revealed PrP deposits in 2 out of 6 muscles (i.e., longissimus dorsi and pectoralis profundus muscles) of the experimentally inoculated animal, and in 4 out of 16 muscles (i.e., trapezius muscle, biceps femoris muscle, semitendinosus muscle and peroneus muscle) of one of the natural asymptomatic BASE cases. Infectivity of the tissues were analyzed by intracerebral inoculation to Tg mice overexpressing bovine PrP (Tgbov XV line^123^). All of 5 mice that were inoculated with the brain homogenate from the experimental BASE animal or from one of the natural BASE cases developed the disease with incubation period of 186 ± 10 days and 178 ± 6 days, respectively. The longissimus dorsi muscle from the experimental BASE case caused the disease in 5 out of 7 mice with incubation period of 380 ± 11 days. The intercostalis and gluteus muscles from the natural BASE case also caused the disease in one out of 7 and 9 mice, respectively, with incubation period of 370 and 498 days, respectively. Thus, PrP^Sc^ was detected in muscles of BASE cases at the terminal stage and asymptomatic stage, but infectivity of the muscles was clearly lower compared with that of the brain, and much lower in asymptomatic than symptomatic animal. Infectivity was not detected in the spleen, lymph node and kidney^124^^)^.

Three calves aged 3 to 4 months were intracerebrally inoculated with brain homogenate from a H-BSE case, and killed at 507-598 days post exposure, i.e. 7-10 days after they developed ataxia of the forelimbs and hindlimbs, and myoclonus. PrP^Sc^ was detected in the brain and spinal cord by WB with phosphotungstic acid precipitation and IHC. PrP^Sc^ was detected by the WB method in most of the peripheral nerves, ganglia, optic nerve, retina, hypophysis, and adrenal gland, but intensity of the signal was barely detectable in most of these tissues, indicating low levels in peripheral nerves even in symptomatic cattle^125^^)^.

Since PrP^Sc^ was not detected in peripheral tissues by conventional IHC, the authors tried to detect it by using IHC with highly sensitive biotinylated tyramide-based procedure. In addition to wide distribution of PrP^Sc^ in the brain and spinal cord, positive PrP^Sc^ immunolabeling was detected in the retina, neurohypophysis, optic nerve, adrenal gland, cauda equina, cervical spinal nerves, facial nerve, hypoglossal nerve, vagus nerve, sciatic nerve, and ganglia such as the inferior ganglion of the vagus nerve, ganglia of the sympathetic trunk, superior cervical ganglion, stellate ganglion, and celiac and mesenteric ganglion complex. However, immunolabeled PrP^Sc^ was not detected in the enteric nervous system such as the myenteric and submucosal plexi of the digestive tract as well as lymphoid tissues^126^^)^.

Five calves that were intracerebrally inoculated with homogenates of the medulla oblongata of a L-BSE cow were killed before and after the onset of clinical signs at 10- and 12-months post exposure, respectively, and at 16 months post exposure in the terminal stage of disease. PrP^res^ was detected by WB in all obex samples. In the preclinical animal, PrP^res^ was detected in the spinal cord, cauda equina, optic nerve, pituitary gland, trigeminal ganglia, cranial cervical ganglia, stellate ganglia, vagosympathic trunk, cranial mesenteric ganglia, and vagus nerve. In the other animals at clinical or terminal stage, PrP^res^ was also detected in other tissues including all or some of the phrenic nerve, accessory nerve, suprascapular nerve, brachial nerve plexus, median nerve, radial nerve, sciatic nerve, tibial nerve, and adrenal gland. However, PrP^res^ was not detected in the facial nerve, hypoglossal nerve, spleen, tonsil, parotid lymph nodes, lateral retropharyngeal lymph nodes, mandibular lymph nodes, brachiocephalic lymph node, anterior cervical lymph node, axillary lymph nodes, superficial inguinal lymph nodes, sub-iliac lymph nodes, popliteal lymph nodes, splenic lymph nodes, hepatic lymph nodes, internal and external iliac lymph nodes, and mesenteric lymph nodes from all the animals^127^^)^. However, based on the result of bioassay for infectivity of the tissues using Tg mice overexpressing bovine prion, infectious titers of peripheral nerve tissues of the calves were estimated to be 1,000-fold lower than those of the obex regardless of clinical stage^127^^)^, indicating low risks of the peripheral tissues.

One EK211 calf and one wild-type calf at less than 2 months of age were intracranially inoculated with homogenate of the brain from an atypical BSE case possessing the E211K PRNP polymorphism. The inoculated EK211 and wild-type calves were killed at 9.8- and 18.1- months post exposure, respectively, when they showed clinical signs. Tissues analyzed by IHC were the liver, kidney, spleen, skin, striated muscles, thyroid gland, turbinate, trachea, lung, esophagus, rumen, reticulum, omasum, abomasum, small intestine including the ileum, adrenal gland, pancreas, urinary bladder, lymph nodes, tonsils, pituitary gland, trigeminal ganglion, brain (cerebral cortex, cerebellum, midbrain, including superior colliculus, brainstem including obex), spinal cord (cervical, thoracic, lumbar), and eye. The analysis showed that the brain, spinal cord (cervical, thoracic, and lumbar), and retina were positive for PrP^Sc^, whereas the other tissues were negative^128^^)^.

In addition to very low or undetectable levels of PrP^Sc^ in peripheral tissues including muscles of L- and H-BSE cattle, a possible difference in gastrointestinal degradation between atypical BSE PrP^Sc^ and C-BSE PrP^Sc^ should be taken into account in risk assessment of atypical BSE for food safety: L-BSE PrP^Sc^ and H-BSE PrP^Sc^ have been observed to be less resistant against PK digestion than C-BSE PrP^Sc^^41^^,^^43^^,^^129^^)^, indicating that atypical BSE PrP^Sc^ could be digested by gastrointestinal enzymes more readily than C-BSE PrP^Sc^.

## 8. The Occurrence of BSE in Cattle and BSE Control Measures Employed in Japan

**Fig. 1:**
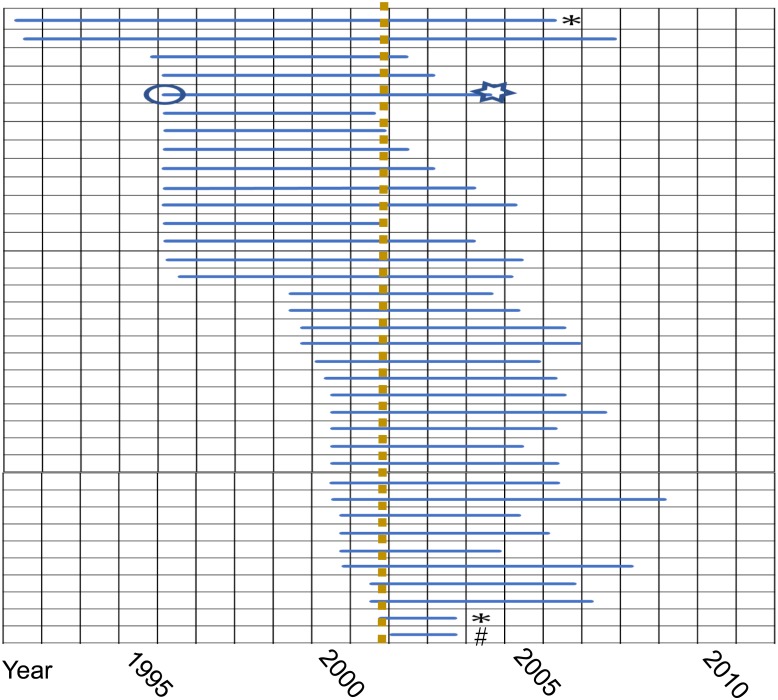
Individual BSE cases confirmed in Japan. Each horizontal bar (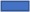) indicates the period between the birth and death (including slaughter) of each case. 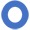
: Birth,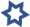: Death, *:L-BSE case, #: The brain tissue has no infectivity to Tg mice overexpressing bovine PrP. 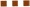
: The time of the ban of manufacturing and sale of the feed for livestock con

In Japan, 36 cases of BSE were identified during the period from 2001 to 2007 (Fig. 1). Among the 36 confirmed cases, two were diagnosed as L-BSE. One was detected in 2006 at the age of 169 months, and the other was in 2003 at the age of 23 months. No clinical signs were observed in the latter 23-months old case. PrP^Sc^ deposits in the obex were scarce and were estimated to be approximately 1/1,000th of those seen in cases of C-type BSE. cases. Moreover, no infectivity was observed in the obex by bioassay using TgBovPrP mice overexpressing bovine PrP^64^^,^^66^^)^.

The latest-born BSE case, which was born in January 2002 and diagnosed as BSE at the age of 21 months. It was suspected that the animal was fed with contaminated feed that had been sold before implementation of the complete feed ban. The accumulation of PrP^Sc^ in the medulla oblongata at the level of obex was estimated to be approximately 1/1,000th that seen in the other C-type BSE case, and no infectivity was observed in the brainstem by bioassay using TgBovPrP and ICR mice^64^^)^.

Table 1 shows chronology of control measures for BSE in Japan. Before 1996, the BSE control measures employed in Japan involved the strengthening of import conditions for meat products and MBM from BSE-occurring countries and a ban of the importation of live cattle from the UK. In 1996, the passive surveillance of BSE with clinical and pathological examinations began legally based on designation of BSE as a notifiable disease according to a Cabinet Order of the Act on Domestic Animal Infectious Diseases Control, and as a disease to be examined at slaughterhouses under the Abattoir Act. From this time, regulatory activity relating to the import of ruminant-derived products were increased, but this mostly involved the provision of guidance with weak legal force.

**Table 1:** Chronology of control measures for BSE in Japan

1990	
July;	-Ban of importation of live cattle from UK
1991	
April;	-Strengthening of import conditions for meat products and MBM from Switzerland
Sep.;	-Strengthening of import conditions for heat-treated meat and MBM from France
1992	
Sep.;	-Strengthening of import conditions for meat of Artiodactyla animals and MBM from Denmark
1994	
Feb.;	-Strengthening of import conditions for bovine semen and MBM from Germany
1995	
March;	-Strengthening of import conditions for heat-treated processed meat and MBM from Italy
1996	
March;	-Guidance of voluntary restriction of import of beef (including offal and bone) and beef products from UK
	-Suspension of import of beef products and MBM from UK
April;	-Guidance of disposition of bovine offal which had been imported from UK
	-Guidance not to import the feed and pet-foods from ruminants produced in UK
	-Guidance of prohibition of the use of MBM of ruminant origin for ruminant feed.
	-Guidance of prohibition of the use of substances derived from ruminants produced in UK as materials of veterinary medicinal products
	-Confirmation of the countries producing the materials of beef products imported from any countries to avoid importation of the beef products of UK via other countries. Voluntary restriction of the beef products which were confirmed to contain materials of UK origin.
	-BSE was designated as a notifiable disease by the Cabinet Order of the Act on Domestic Animal Infectious Diseases Control
	-BSE was designated as a disease to be examined at abattoirs under the Abattoir Act; -Starting of the surveillance based on clinical and pathological examinations.
1997	
March;	-Adoption of the supplementary resolution at the House of Representatives and Councilors on the guidance for prohibition of the use of MBM derived from cattle and sheep for these animals
April;	-BSE was designated as a notifiable disease by the Act on Domestic Animal Infectious Diseases Control
2000	
Dec.;	-Guidance of voluntary restriction of the import of the cattle brain and spinal cord of EU country origin
	-Guidance for thorough dissemination of the information that the feed materials (MBM etc) including ruminant tissues are not to be used for feed
	-Suspension of the import of the processed animal proteins such as MBM from EU countries
	-Guidance of voluntary restriction of the import of beef (bovine meat and offal, and their products)
2001	
Feb.;	-Ban of the importation of bovine meat and offal, and meat products produced from them, from countries and regions with BSE occurrence.
	-Guidance for voluntary restriction of the bovine meat etc imported via EU countries
April;	-Surveillance guideline to strengthen BSE testing using Western blot
	-Start of the surveillance based on testing the presence of abnormal prion proteins in “cattle at or over 24 months of age that are suspected to have nervous symptoms such as a consciousness, movement, sensory, reflection, or other disorders”
June;	-Guideline on the prevention of the contamination of feed for ruminants with proteins of ruminant origin
Sep.;	<The first case of BSE was identified>
	-Ban of the production, sale, and use of the protein derived from cattle and other ruminants for cattle feed under the Act on Safety Assurance and Quality Improvement of Feeds
	-Strengthening of BSE screening test at abattoirs for; all the cattle at or over 24 months of age that show general symptoms and are suspected to have nervous symptoms such as a consciousness, movement, sensory, reflection, or other disorders; all the cattle at or over 30 months of age including the cattle without suspected nervous symptoms
	-Notification to prefectural governments of strengthening the surveillance at farms (testing and burning of the cattle showing central nervous symptoms)
	-Guidance to relating businesses via prefectural governments on the removal and burning of the skull (excluding tongue and cheek meat) of cattle at or over 12 months of age and the spinal cord and distal ileum (two meters from the connection to the cecum) of cattle at all ages
Oct.;	-Request of temporary prohibition against the importation, production and shipping of MBM (including MBM derived from poultry and pigs) for feed and fertilizers to feed manufacturers via prefectural governments and relating groups (implement from Oct. 4)
	-Ban of manufacturing and sale of the feed for livestock containing MBM, and feeding livestock with the feed, under the Act on Safety Assurance and Quality Improvement of Feeds (the date of implementation is the day of legislation; the date of implementation is Nov. 1 for the feed that had been manufactured and shipped prior to the legislation)
	-Partial amendment of the Ordinance for Enforcement of the Slaughterhouse Act; Obligation of burning of the head (excluding tongue and cheek meat), spinal cord, and two meters of the ileum from the connection to the cecum (proviso; the head is to be read as brain and eyes during one year after the implementation)
	-Start of BSE test (screening test at 117 meat hygiene inspection centers) for all slaughtered cattle
Nov.;	-Introduction of the Ministerial approval system on manufacturing of poultry protein such as poultry meal, and resumption of the use of poultry protein for feed for poultry, swine, and farm-raised fish
	-Resumption of the use of fish meal that is self-checked by fish meal producing plant not to be contaminated with mammalian proteins
Dec.;	-Decision to burn the meat of cattle that had been slaughtered and processed before Oct. 17
2002	
Feb.;	-Ban of the use of fish meal for cattle feed
August;	-Strengthening of the standard of animal oil/fat: For calf milk replacer, they must be produced from fat of edible meat and contain insoluble impurities of <0.02% in weight.; Other feed must contain insoluble impurities of <0.15% in weight.
Nov.;	-Inclusion of tonsil in SRM.
2003	
April;	-Strengthening of the standard of animal oil/fat: For ruminant fee except for calf milk replacer, they must be produced from fat of edible meat
June;	-Announcement of the rule of separation of the production lines of the feed for ruminants from those for other animals at feed mills (interim measures until 2005)
Sept.;	-Guideline for the prevention of contamination of the feed for ruminants with animal derived proteins
Oct.;	-Strengthening of the standard of oil/fat originated from ruminants: Ban of the use of oil/fat originated from fallen stock etc for a raw material of feed
Dec.;	-Start of the tracing system for bovine at production sites under the Act on Special Measures on the Management and Relay of Information for Individual Identification of Cattle
2004	
Jan.;	-Introduction of the Ministerial approval system on fish meal manufacturing
Feb.;	-Vertebral column was included in SRM
April;	-Mandatory BSE test for all dead cattle at 24 months and over
May;	-Implementation of the ban on feed and fertilizer to avoid contamination with cattle vertebral column i.e., the ban of the use of animal fat derived fallen stock cattle for ruminant feed, the use of animal fat derived from cattle vertebral column and fallen stock cattle for pig and poultry feed, and the use of anything derived from cattle vertebral column fallen stock cattle for fertilizer,
	-Requirement of insoluble impurities of <0.02% in weight for the ruminant fat used for cattle feed
Sep.;	-Interim report on the comprehensive review of the measures on BSE in Japan by the Food Safety Commission
Dec.;	-Start of the tracing system for bovine at market distribution sites under the Act on -Special Measures on the Management and Relay of Information for Individual Identification of Cattle
2005	
April;	-Implementation of separation of the production lines of the feed for ruminants from those for other animals at feed mills
	-Introduction of the Ministerial approval system on porcine protein manufacturing and resumption of the use of the protein for swine and poultry feed
May;	-”Risk Assessment Related to Measures against Bovine Spongiform Encephalopathy (BSE) in Japan” by the Food Safety Commission (including the risk associated with a change of cattle for BSE testing at abattoirs from the cattle of all ages to that over 20 months of age)
July;	-Notification of the change of cattle for BSE testing at abattoirs from the cattle of all ages to that over 20 months of age
Dec.;	-”Risk assessment concerning the comparability between risks of consuming cattle meat and offal regulated by the beef export verification program of the United States/Canada and risks of consuming cattle meat and offal of Japanese cattle” by the Food Safety Commission
2008	
May;	- Resumption of the use of the swine MBM produced by approved rendering plants for farm-raised fish feed
2009	
April;	- Ban of pithing at abattoirs
2012	
Oct.;	- “Consideration of Risk Variations in Japan Derived from the Proposed Revisions of the Current Countermeasures against BSE” by the Food Safety Commission
Nov.;	- “Risk assessment for revision of the Standards and Criteria for Food and Food Additives on the vertebral column”
2013	
Feb.;	
	-Notification of verification program for beef and beef products from the USA, Canada, France, and the Netherland.
Jun;	-Notification of the change in cattle age for BSE testing for healthy slaughtered cattle at abattoirs at abattoirs from over 30 months of age to over 48 months of age
Dec.;	- Notification of verification program for beef and beef products from Ireland
2014	
Jan.;	-Resumption of the use of cattle MBM (containing no SRM) for fertilizer (not to be used in pastures) which is mixed with repellents or chemical fertilizer not to be fed by ruminants
August;	- Notification of verification program for beef and beef products from Poland
2015	
March;	-Resumption of importation of gelatin and collagen produced from bovine skin, and their products
April;	-Resumption of the use of cattle MBM (containing no SRM) produced by approved rendering plants for farm-raised fish feed
Dec.;	- Notification of verification program for beef and beef products from Brazil
2016	
Feb.;	- Notification of verification program for beef and beef products from Norway, Denmark, Sweden
May;	- Notification of verification program for beef and beef products from Italy
July;	- Notification of verification program for beef and beef products from Switzerland and Lichtenstein
August;	- “Risk assessment for revision of the domestic BSE countermeasures (on cessation of BSE testing for healthy slaughtered cattle)” by the Food Safety Commission
2017	
Feb.;	-Notification of the cessation of BSE testing for healthy slaughtered cattle; continued implementation of BSE testing for the cattle (>24 months) which are judged by ante mortem inspection to have systematic symptoms or suspected nervous symptoms
Sep.;	- Notification of verification program for beef and beef products from Austria

The first BSE case identified in Japan was a cow slaughtered in August 2001^130^^,^^131^^)^. A series of control measures with binding legal force were taken later in 2001, including a ban on the import of MBM, a ban on the use of MBM for feed of all animals (designated as complete feed ban), the burning of specific risk materials (SRM) (the head excluding tongue and cheek meat; the spinal cord; the ileum of 2 meters long from its connection with the cecum). The tonsil and the vertebral column were included in the list of SRM in 2002 and 2004, respectively, and all the cattle slaughtered at slaughterhouses were subjected to screening tests for BSE from this point onwards^132^^)^. In 2004, mandatory BSE test was initiated for all cattle died over the age of 24 months, and the tracing system for bovines throughout market distribution was introduced under the Act on Special Measures Concerning the Management and Relay of Information for the Individual Identification of Cattle. Based on a risk assessment by the Food Safety Commission of Japan (FSCJ), the age of cattle to be tested for BSE at slaughterhouses was changed from “all ages” to “over 20 months of age” so that only cattle over 20 months of age had to be tested for BSE at slaughterhouses.

After being requested by the Ministry of Health, Labor and Welfare (MHLW) in 2011 on revision of the countermeasures against BSE in Japan, the FSCJ conducted a risk assessment focusing on the age limit of cattle for BSE testing and the definition of SRM (the skull excluding tonsils, and the spinal cord and vertebral column) relating cattle age, and published a report in 2012. In this assessment, in view of the status of the occurrence of BSE in Japan after the tightening of the feed-control regulations in October 2001, the amount of the BSE agent fed by a head of cattle in Japan was estimated not to exceed the amount contained in 1 g of brain material of field BSE cases identified in the UK. Among the cattle that were orally inoculated with 1 g of the brain tissues from the UK BSE cases, clinical signs and PrP^Sc^ in the CNS were initially detected at 44 months post exposure. However, PrP^Sc^ was not detected in the CNS at 42 months post exposure; i.e., over 46 months of age^24^^,^^31^^)^. Furthermore, studies of intracerebral inoculation of BSE-infected materials into cattle demonstrated that PrP^Sc^ was first detected in the brainstem at 7–8 months before the onset of clinical signs. Based on these findings, the FSCJ considered that the possibility for PrP^Sc^ to be detected in the CNS is extremely low in cattle aged below 30 months.

As for vCJD, its incidence in the UK peaked in 2000, but then gradually declined. The OIE has reported its global incidence also markedly decreased from its peak to only several cases per year up to 2016, December 31. Due to the close link between yearly incidence of vCJD and that of BSE, the FSCJ concluded that the regulatory actions taken in Japan including prohibition of the use of SRMs had effectively reduced the risk of infection with the BSE agent in both cattle and humans.

Regarding atypical BSE, most cases were found in cattle aged over 8 years (range: from 6.3 to 18 years). Although one case was detected at the age of 23 months in Japan, the medulla oblongata tissue from this case did not show transmissibility in highly susceptible Tg mice. According to this finding, the FSCJ considered that the cattle younger than 8 years pose negligible risk on human infection with the atypical BSE agent.

Thus, based on the BSE status and infection risk to cattle in Japan, and the interspecies barrier to BSE transmission between cattle and humans, the FSCJ evaluated that vCJD is highly unlikely to develop through consumption of meat and offal (excluding the tonsils and distal ileum) from cattle aged at or below 30 months under the continual implementation of the feed control measures. Therefore, the FSCJ concluded that the change in the age limit for BSE testing of cattle from 20 months to 30 months and the change in the definition of SRM (the skull excluding tonsils, and the spinal cord and vertebral column) from “in cattle at all ages” to “in cattle aged over 30 months” causes a negligible influence on human health^133^^)^.

The FSCJ conducted a further risk assessment of the age limit for BSE testing in cattle and published a report in 2013. In the report, The FSCJ proposed that vCJD is highly unlikely to occur through consumption of meat and offal (excluding SRM) derived from the cattle born and raised in Japan. This was based on the status of occurrence of BSE in cattle and the implementation of control measures, such as import restrictions, feed restrictions, and appropriately performed processing at slaughterhouses, in Japan. According to this report, if no BSE cases were detected among the cattle that were born before 11 years ago, the incidence of BSE in these birth cohorts would be negligible as far as the control measures against BSE are continuously implemented, because the number of BSE cases detected by the BSE surveillance in the EU^134^^)^ suggested that most BSE-infected cattle—approximately 97%—can be detected before the age of 11 years. In Japan, no BSE cases had been identified among cattle born between January 2002 to May 2013. Therefore, BSE was highly unlikely to occur in the birth cohort born in February-December of 2002.

However, viewing the difficulty of prediction of BSE incidence in younger cohorts born after 2002, the FSCJ concluded that the age limit for BSE testing could be raised tentatively from 30 months to 48 months to verify the efficiency of the control measures in the cohorts born after 2002^135^^)^.

Based on the results of these two risk assessments by the FSCJ, the MHWL notified the cattle age of BSE testing at slaughterhouses to be raised from over 20 months to over 30 months in February 2013, and then from over 30 months of age to over 48 months in June 2013.

In 2016, in response to the request by the MHLW for a risk assessment, which was required to revise the countermeasures against BSE in Japan, the FSCJ conducted another risk assessment on the age limit for the BSE testing in cattle.

Based on the status of the occurrence of BSE after the risk assessment conducted in 2013, the FSCJ considered that C-type BSE was most unlikely to occur during the continuous implementation of the control measures for BSE including feed restrictions.

Regarding atypical BSE, the available findings on the experimental transmission of H-BSE to laboratory animals suggested that the animal-to-human transmission is unlikely to occur. Although brain tissue from L-BSE cattle possibly causes human infection, the infectivity of the tissues other than SRM was estimated to be very low. Furthermore, the incidence of L-BSE in cattle was very low in Japan and the EU (0.07 and 0.09/1,000,000 heads, respectively), and no epidemiological association has been detected between atypical BSE and human diseases. Thus, the FSCJ considered that vCJD is unlikely to occur through the consumption of meet or offal (excluding SRM) derived from the cattle born and raised in Japan. Based on this, the FSCJ concluded that there is no concern about human health regardless of whether BSE testing at slaughter will continuously be implemented for cattle aged over 48 months or will be ended for cattle at any age.

The FSCJ, however, emphasized the importance of feed regulation, continuous monitoring of high-risk cattle, and ante-mortem inspection at slaughterhouses. In addition, the FSCJ suggested that appropriate BSE testing is needed for the cattle aged over 24 months that are suspected by inspection to have impairments of nervous system such as ataxia, paresthesia, dysreflexia, and impaired consciousness, or systematic symptoms.

In February 2017, the MHLW notified to end the BSE testing for healthy slaughtered cattle. Since April 2017, the active surveillance for BSE in healthy cattle at slaughterhouses has not been performed. However, BSE testing has continuously been performed at slaughterhouses for the cattle aged over 24 months of age, when they are judged by ante-mortem inspection to have systematic symptoms or the suspected nervous symptoms. For cattle that died at farms, the age of BSE testing was raised from over 24 months to over 48 months in April 2017.

The feed-control measures including disposal of SRM have continuously been implemented. Only one BSE case was identified among the cattle born in January 2002; i.e., soon after the introduction of a series of the feed-control measures (the complete feed ban) in 2001, demonstrating that the complete feed ban was highly effective at preventing the occurrence of BSE in cattle in Japan. In agreement with this notion, a stochastic model study showed that the three interventions (SRM removal, post-mortem testing and cohort culling) reduced the risk of BSE by 98.95% from 2002 to 2009^136^^)^.

In contrast, the incidence of BSE in cattle born before 2001 indicated that the control measures taken before 2001 were not effective enough to prevent BSE in Japan. In fact, live cattle had been imported from the U.S.A. and Canada, MBM were imported from Italy and Denmark, and animal fat was imported from the Netherlands, during the period from April 1996 to September 2001. In addition, SRM had continued to be used for rendering, and feed had been produced under conditions in which cross-contamination between cattle and other livestock feeds might have occurred. Furthermore, MBM had been used for a supplement in many dairy farms in Japan^137^^)^. Thus, comparative effectiveness of the risk management measures taken by the Japanese government in the periods before and after the identification of the first BSE case in 2001, if analyzed, could provide a valuable basis for decision-making in the field of food safety.

## 9. Born after the Reinforced Ban (BARB)

In Great Britain, the use of mammalian meat and bone meal (MMBM) for farm animal feed was prohibited by the reinforced legislation that was introduced on August 1, 1996, to prevent further exposure of cattle to the BSE agent. The analysis of BSE cases (BARB cases) that were born after implementation of this additional control measure and were detected before December 31, 2008, showed no evidence of the involvement of a maternal risk factor, infection from environmental contamination (other than from feedstuffs) and a genetically based etiology. The epidemiological features were consistent with the involvement of an exogenous feed-borne source due to reliance on imported feedstuffs in Great Britain and the later introduction of a ban of the use of MBM in other EU member states^138^^)^.

The case-control study based on data of the BARB cases (totally 164 cases) that were detected in Great Britain until July 31, 2009, showed that the cases were exposed to “homemix” (compound feed mixed on farms) or a combination of “homemix”. There was no evidence that indicated an involvement of environmental contamination in infection^139^^)^.

The descriptive analysis of BSE cases in Ireland suggested “feeding of concentrates” as the only common factor to all BARB cases for which information existed. Clustered spatial pattern and dairy herd type in the Irish BARB cases suggested that the BARB cases did not arise spontaneously but rather arose through consumption of contaminated feed^140^^)^.

In the EU, the number of BARB cases in each birth cohort declined exponentially after introduction of total feed ban until 2016^141^^)^. To find the source of infection in BARB cases in EU member countries, the EFSA Panel on Biological Hazards (BIOHAZ) et al reviewed data of 60 BARB cases that were born after the enforcement of the EU total feed ban. The review suggested that feed-borne exposure was the most likely source of infection compared with the other possible sources, although the source in each case remained mostly uncertain^142^^)^.

The finding of the absence of detectable PrP^Sc^ in urine and milk of infected cattle suggested that these fluids were not the source of infection^57^^,^^73^^,^^143^^)^.

One of possible sources is the manure derived from the cattle that ingested a large amount of BSE PrP^Sc^. Cattle manure is known to contain proteins, indicating that undigested proteins are excreted in feces in cattle. Based on *in vitro* studies, Böhnlein et al suggested that BSE PrP^Sc^ survives during gastrointestinal digestion^40^^)^. Therefore, the prions ingested by cattle are likely to be excreted in part in their feces and contaminate environments surrounding the cattle. This is consistent with the findings of fecal excretion of PrP^res^ in the mice and coyotes that were exposed orally to brain homogenates from BSE and CWD animals, respectively^144^^,^^145^^)^.

Scrapie prions have been observed to persist in soils for years. For BSE prions, Maddison et al^146^^,^^147^^)^ showed highly efﬁcient binding of bovine BSE PrP^Sc^ to different types of soil within 24 h. Clay-rich soils induced N-terminal truncation of the PrP^Sc^, but sand-rich soils yielded full length PrP^Sc^ species. The recovery and persistence of PrP^Sc^ in sandy loam soil decreased during the 18-months incubation period in a temperature-dependent manner, but average percent of recovery at day 566 remained over 10% of that at day 1-4 at any temperatures.

Binding of TSE prions with soil particles may keep the prions near the surface of soil, and thereby may increase a chance of animal exposure. Consistent with this is the observation that soil particle-associated hamster-adapted 263K scrapie prions and transmissible mink encephalopathy agent caused infection in hamsters^148^^)^.

A study of uptake of CWD prions by wheat (*Triticum aestivum*) showed no detectable PrP^Sc^ in the stem of the plant after its root was exposed for 24 hr to the brain extracts from CWD-positive elk, indicating that the PrP^Sc^, if transported from the root to the stem, was at the level below the limit of detection of diagnostic WB and other test kits^149^^)^. However, Pritzkow et al reported that plants were contaminated with PrP^Sc^ from soil. They grew barley grass plants (*Hordeum vulgare*) on soil that had been contaminated with 263K hamster brain homogenates. After grown for 3 weeks, pieces of stem and leaves of the plants were analyzed for the presence of PrP^Sc^ by PMCA. All the plants grown on the contaminated soil contained PrP^Sc^ in their stem although in small quantities. Moreover, one of the four plants contained PrP^Sc^ in their leaves^150^^)^.

Thus, we cannot fully neglect that ruminants could be exposed to BSE PrP^Sc^ through ingestion of grasses that are contaminated with PrP^Sc^ from soil. Contamination of grass could also pose a risk, if fertilizers used for grass are contaminated with PrP^Sc^. After being ingested by animals, PrP^Sc^ may be diluted during spreading from the gastrointestinal tract to feces, and then to soil and grass. Therefore, grazing the grass contaminated with PrP^Sc^ seems unlikely to be the major risk factor in BSE infection in cattle, but it could accidentally pose an un-ignorable risk of BSE infection in ruminants.

Persistence of PrP^Sc^ in environments for long period has also been observed in sewage. A study of the fate of BSE PrP in sewage showed that PrP^res^ decreased to the level undetectable by immunoblotting after 150 days of incubation, whereas infectious titer in mice did not decrease during this period. Likewise, persistence of infectious titer was observed in PrP^Sc^ incubated with PBS until 265 days of incubation^151^^)^. Taken together with possible contamination in soil, this study has suggested that the environmental contamination could become an un-ignorable risk for the occurrence of BSE in cattle under insufficient risk managements for inactivation and decontamination of PrP^Sc^.

## 10. Conclusion and Further Research Need

The decrease of incidence of BSE around the world following the implementation of feed-control measures indicated that the BSE epidemic was directly caused by feeding cattle with MBM containing BSE PrP^Sc^. The subsequent decrease in the annual number of vCJD cases is attributable to the decrease in the number of BSE cattle itself as well as the implementation of the control measures intended to exclude the BSE agent from human food. Thus, a series of the feed- and food-control measures including the measures for prevention of re-circulation of BSE PrP^Sc^ were effective at reducing the incidence of BSE and vCJD. The control measures might have lowered also the risk of human exposure to the BSE PrP^Sc^ that derived from small ruminants^152^^,^^153^^)^.

In Japan, 36 cases of BSE were identified during the period from 2001 to 2007. During this period, feed-control measures including the disposal of SRM have continuously been implemented. Among the cases, two were diagnosed as L-BSE. The latest-born BSE case, which was born in January 2002, was diagnosed as C-BSE at the age of 21 months soon after the introduction of a series of the feed-control measures (the complete feed ban) in 2001. The data demonstrated that the complete feed ban was highly effective at preventing the occurrence of BSE in cattle in Japan as was the case in EU countries.

As for BARB cases, which were detected in several countries, feed-borne exposure has been regarded the most likely source of infection compared with the other possible sources.

Studies of oral administration of the C-BSE agent in cattle at their age less than 1 year shed light on the dose-dependent timing of appearance of clinical symptoms and PrP^Sc^ accumulation in the CNS. Massive doses of the brain tissue from C-BSE cases induced PrP^Sc^ accumulation in the CNS from 30 months post exposure and clinical signs from 35 months with PrP^Sc^ accumulation in the PNS. Lower doses induced PrP^Sc^ accumulation in the CNS and clinical signs at 44 months post exposure.

The brain, spinal cord, DRG, and ileum were the major locations of PrP^Sc^ accumulation in orally infected C-BSE cattle. Infectivity was higher in the ileum than in the other portions of small intestine regardless of dose. PrP^Sc^ was detected in IPP from 2 months after oral exposure, being concentrated in CPP rather than DPP. Age-dependent changes in numbers of lymph follicles and PrP^Sc^-positive lymph follicles in the ileum imply the decrease in intestinal PrP^Sc^ with the time elapsed after oral exposure,

PrP^Sc^ was detected in the muscle in clinically affected C-BSE cattle, but not in preclinical cattle. A small amount of PrP^Sc^ deposits was also detected in muscle spindle, although its contribution to the amount of PrP^Sc^ in the whole of muscles is unclear. No infectivity was noted in fat tissues from the cattle orally inoculated with the brainstem of BSE-affected cattle.

The sporadic nature of H-BSE and L-BSE occurrence suggested that the atypical BSE may occur spontaneously rather than by feed-mediated infection. Higher zoonotic potential of L-BSE than C-BSE was suggested by some experimental studies, but the reverse was also observed by other studies. Calves were more susceptible to C-BSE than L-BSE, but the reverse was observed in non-human primates although based on a limited number of animals. Zoonotic potential of H-BSE has been suggested to be lower than that of C-BSE.

Features of tissue distribution of PrP^Sc^ in naturally or experimentally infected atypical BSE cattle resembled to those in C-BSE cattle except in the intestine, where PrP^Sc^ accumulation was not observed in the atypical BSE. PrP^Sc^ was detected in the muscle and peripheral nerves in atypical BSE cattle, but the levels were much lower than those in the CNS.

Thus, experimental and epidemiological findings accumulated during the past three decades shed light on characteristic features of BSE, and thereby led the successful reduction of BSE. However, from the perspective of food safety, unresolved issues remain even in light with the accumulated findings. The issues are as follows: a) The relative contribution of each control measure to the reduction of BSE remains mostly unclear, although the contribution was estimated for intervention strategies such as SRM removal, post-mortem testing for BSE, and culling of BSE cases in Japan^136^^)^ and the Netherlands^154^^)^. The estimation of relative effectiveness of other control measures is required for precise policymaking including relaxation of the regulations and rules relating to BSE in a risk-based scientifically sound manner. ; b) The ingested C-BSE PrP^Sc^ was stable in the intestinal lumen, but this remains uncertain for atypical BSE PrP^Sc^, which was more sensitive against PK digestion than C-BSE PrP^Sc^. ; c) Taken together with the findings in sheep, susceptibility of calves to oral C-BSE PrP^Sc^ seems likely to decrease with the increase in their age in association with the reduction of the IPP-mediated transfer of PrP^Sc^ from the intestinal lumen to the enteric nerves. However, there is no evidence which demonstrated directly the age-dependent change in bovine susceptibility to the BSE agent.; d) Most experiments of oral exposure to the BSE agent in experimental and farm animals have been performed by a single administration, but effects of repeated or long-term administration are unknown.; e) Oral infectivity of the atypical BSE agent in cattle and PrP^Sc^ accumulation in tissues of the orally infected cattle are unclear.; f) Better understanding of the risk of infection of bovine and other mammalian animals with the BSE agent via soil and grasses is required.; g) Regarding tissue distribution of PrP^Sc^ in C-BSE infected cattle, the ileal infectivity after 40 months post exposure and the quantitative significance of PrP^Sc^ deposits in muscle spindle remain uncertain.

The origin of BSE prions is also an unresolved issue. Although various hypothetical views have been presented on the origin of C-BSE^142^^)^, no definitive conclusions have yet been reached. C-BSE prions might have first arisen through a PRNP gene mutation or the post-translational conversion of PrP^C^ in individual cattle, but no evidence for either pathway exists. Among the various possible sources of C-BSE, scrapie PrP^Sc^ has been regarded as a more likely source because of the occurrence of C-BSE after changes in the rendering practices used for production of MBM, which had possibly been contaminated with scrapie PrP^Sc^. However, the brain of sheep that were naturally infected with scrapie during the BSE epidemic in the UK caused a different disease from BSE in experimentally inoculated cattle^155^^)^, suggesting that the scrapie PrP^Sc^ in its original form could not be a candidate for the origin of the BSE epidemic. However, it remains possible that the cattle affected by the epidemic were exposed to the sheep-derived PrP^Sc^ that had been modified physically in the process of rendering.

A number of studies have demonstrated that annealing, PMCA, or shaking can generate PrP^Sc^ or PrP^res^ from purified hamster PrP^C^ or recombinant PrP in cell-free system in the absence of PrP^Sc^ and PrP^res^ as a “seed”^156^^–^^159^^)^, indicating that the spontaneous unintended generation of PrP^Sc^ might occur in the environment under similar physical conditions. Such conditions could be found in heating, sonication, or/and shaking during the burning of cattle on farms, rendering processes, or food processing. Even if only trace amounts of PrP^Sc^ or PrP^res^ were generated in the environment, they could act as seeds for PrP^Sc^ amplification in PrP^C^-rich tissues if they were ingested by animals and humans. Better understanding of the mechanism and origin of generation of BSE PrP^Sc^ may enable more precise risk assessments and pinpoint managements for prevention of food-mediated BSE-derived human diseases.

The BSE PrP^Sc^ is a unique food safety hazard due to its extremely high resistance to chemical and physical treatments despite its zoonotic potential. Accordingly, the physical and chemical treatments, such as acid- and heat-treatments that are used in food processing and cooking to prevent food-borne microbial diseases, are not effective against the BSE PrP^Sc^. Therefore, lowering the risk of human infection with BSE via food has depended on the effort to prevent BSE infection in food-producing animals and the entry of animal tissues that might contain PrP^Sc^ into food chains.

A basic question in the management aimed at prevention of food-mediated vCJD is whether a tolerable level of intake of BSE prions exists or not. For chemical food safety hazards that are not genotoxic, no-observed-adverse-effect levels based on experimental data of feeding studies are widely used to determine tolerable levels for humans^160^^)^. Dose-response relationships have been studied for BSE by experimental oral inoculation of the brain tissues containing the BSE agent to cattle and sheep^24^^,^^25^^,^^161^^)^, but not in other animal species. The dose-response was studied by ic inoculation of the BSE or vCJD agent in mice^57^^,^^162^^)^, but has not been studied by oral inoculation of the agent in experimental animals including mice. In addition to the lack of the data of oral dose-responses in experimental animals, the marked species difference in susceptibility that is recognized as so-called species barriers^163^^)^, makes the extrapolation of animal data to humans difficult.

Furthermore, it seems difficult to find an appropriate end-point among animal responses, because dosed animals could be in subclinical stages throughout their normal life span. Studies of serial passage of the TSE agents demonstrated that TSE-inoculated animals showed no clinical symptoms and/or no detectable PrP^res^ during their normal life span, although they harbored PrP^Sc89,^^164^^–^^166^^)^. The subclinical stage during a lifespan in experimental animals could not directly extrapolated to humans because of large differences in lifespan between animals and humans.

The analysis of data of a large number of scrapie-challenged mice suggested that no safe dose exists in terms of the threshold dose below which the probability of infection is zero^167^^)^, indicating the difficulty of finding the threshold level for the BSE agent as is the case for most infectious pathogens and genotoxic carcinogens. For food safety-threatening microbial hazards, intake-disease relationships based on epidemiological data, if available, have been used in risk assessments conducted as a basis of decision of control measures^168^^)^. Data available for the BSE agent are limited, but a threshold level of intake of the BSE agent in the UK population was estimated in terms of bovine infectious dose, based on the observed number of vCJD cases and the estimated quantity of the BSE agent entered to cattle-derived food^169^^)^. However, the estimated value may have a limitation in its accuracy especially because of unknown variations of human individual susceptibility. Tolerable intake or intake-dependent risk in humans, if known, could be a basis for future development of cost-effective decision of the control measures aimed at reducing the risk of BSE-derived human diseases, and therefore further research into this issue is required.
